# Malignant Peripheral Nerve Sheath Tumor: MRI and CT Findings

**DOI:** 10.1155/2015/241259

**Published:** 2015-10-29

**Authors:** K. O. Kragha

**Affiliations:** Department of Radiology, University of Louisville, 530 S. S. Jackson Street, CCB C07, Louisville, KY 40202, USA

## Abstract

This is a case report of a 56-year-old male with hypertension who presented with urinary retention and bowel incontinence. CT and MRI of the abdomen and pelvis showed a large complex cystic and solid enhancing mass in the right presacral space. Pathology biopsy result showed malignant peripheral nerve sheath tumor (MPNST) with extensive necrosis. The diagnosis of MPNST is extremely difficult due to the lack of (1) conclusive immunohistochemistry or unique chromosomal anomaly, (2) universal distinctive histopathology, and (3) clinical criteria. The clinical, radiologic, and histologic presentation of MPNST is important in its diagnosis. A rare case of MPNST that produced urinary retention and bowel incontinence is presented that may aid clinicians in the diagnosis of this rare clinical entity. Motor weakness, central enhancement, and immunohistochemistry may assist in the diagnosis of MPNST and differentiation between benign peripheral nerve sheath tumor (BPNST) and MPNST.

## 1. Case Report

This is a 56-year-old male with history of hypertension and neurofibromatosis who presented with sudden onset of urinary retention and bowel incontinence.

## 2. Results

**Figure 1:**
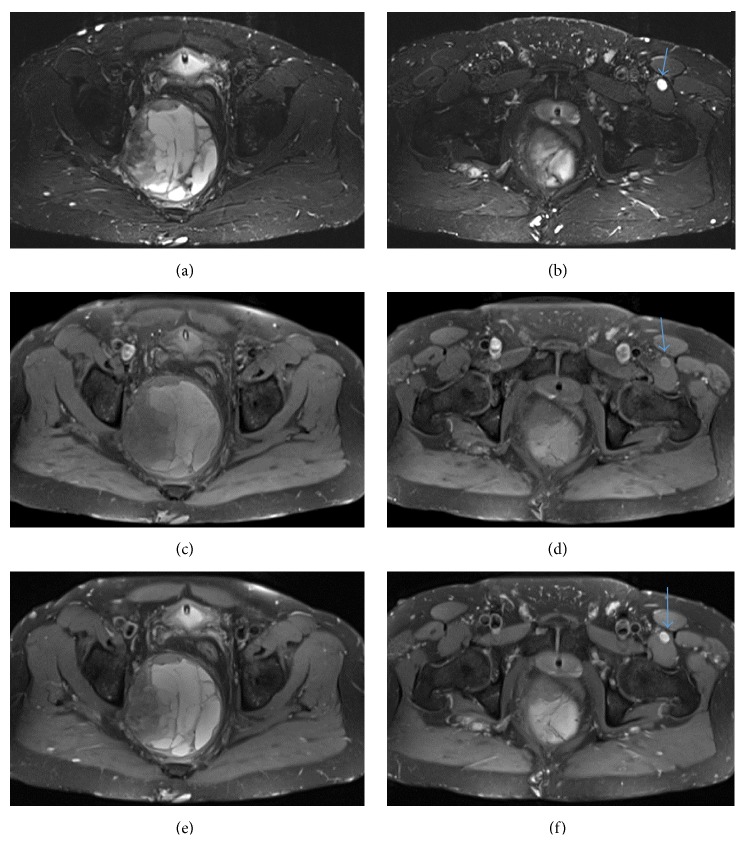
(a) Axial STIR shows pelvic mass. (b) Axial STIR shows pelvic left vastus medialis/intermedius muscle fibroma. (c) Axial T1 FS pelvic mass. (d) Axial T1 FS left vastus medialis/intermedius muscle fibroma. (e) Axial PD FS shows pelvic mass. (f) Axial PD FS shows left vastus medialis/intermedius muscle fibroma.

**Figure 2 fig2:**
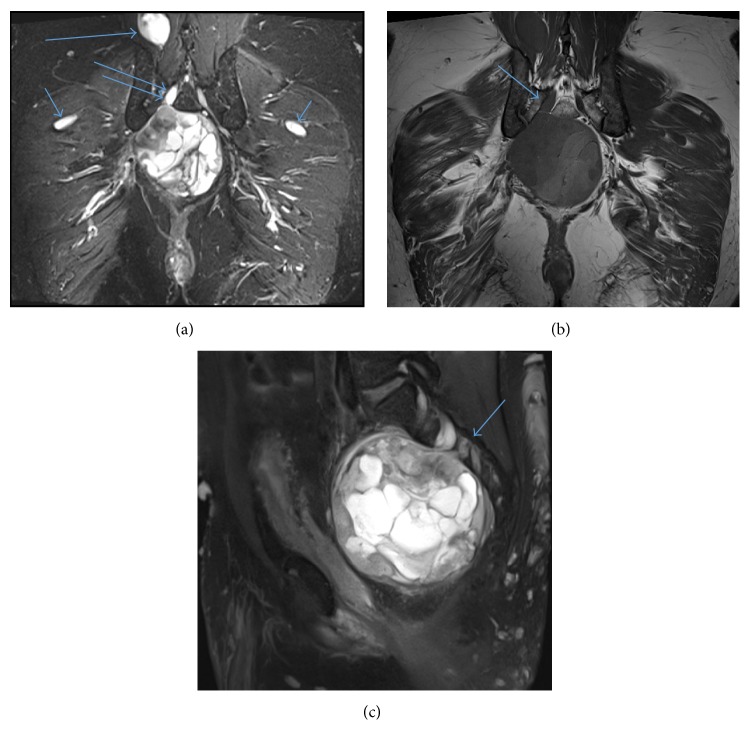
(a) Coronal STIR shows pelvic mass extending to the right S2 neuroforamen (long double arrows), right erector spinae/multifidus muscle neurofibroma (long big arrow) and small bilateral hip neurofibromas (short small arrows). (b) Coronal T1 shows pelvic mass extending to the right S2 neuroforamen (arrow). The other neurofibromas seen in (a) are not well visualized because their signal intensity is isointense to muscle. (c) Sagittal T2 FS shows pelvic mass (arrow points to right S2 neuroforamen).

**Figure 3 fig3:**
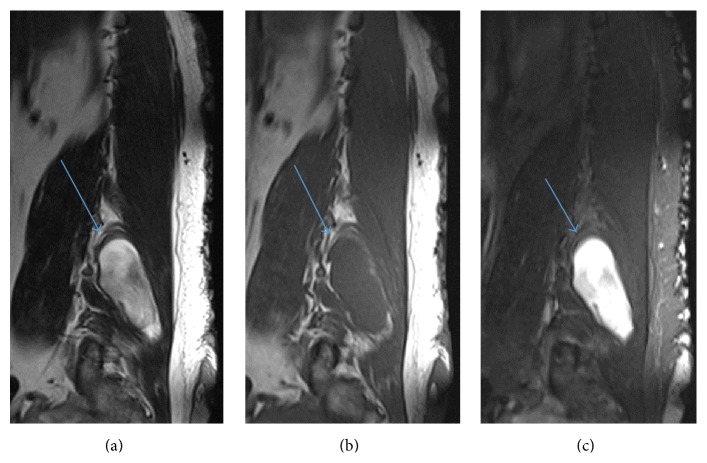
(a) Sagittal T2 shows right erector spinae/multifidus muscle neurofibroma (arrow). (b) Sagittal T1 shows right erector spinae/multifidus muscle neurofibroma isointense to muscle (arrow). (c) Sagittal STIR shows right erector spinae/multifidus muscle neurofibroma (arrow). The neurofibromas on the skin surface are bright on STIR but not well seen on T2 (a) and T1 (b).

**Figure 4 fig4:**
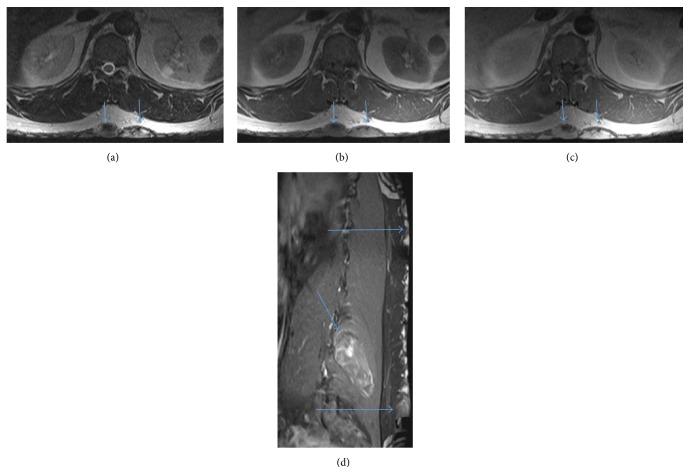
(a) Axial T2 shows neurofibromas on the skin surface (arrows). (b) Axial T1 shows neurofibromas on the skin surface (arrows). (c) Axial T1 + contrast shows enhancing neurofibromas on the skin surface (arrows). (d) Sagittal T1 FS + contrast shows heterogeneous enhancing right erector spinae/multifidus muscle neurofibroma (short big arrow) and enhancing neurofibromas on the skin surface (long thin arrows).

**Figure 5 fig5:**
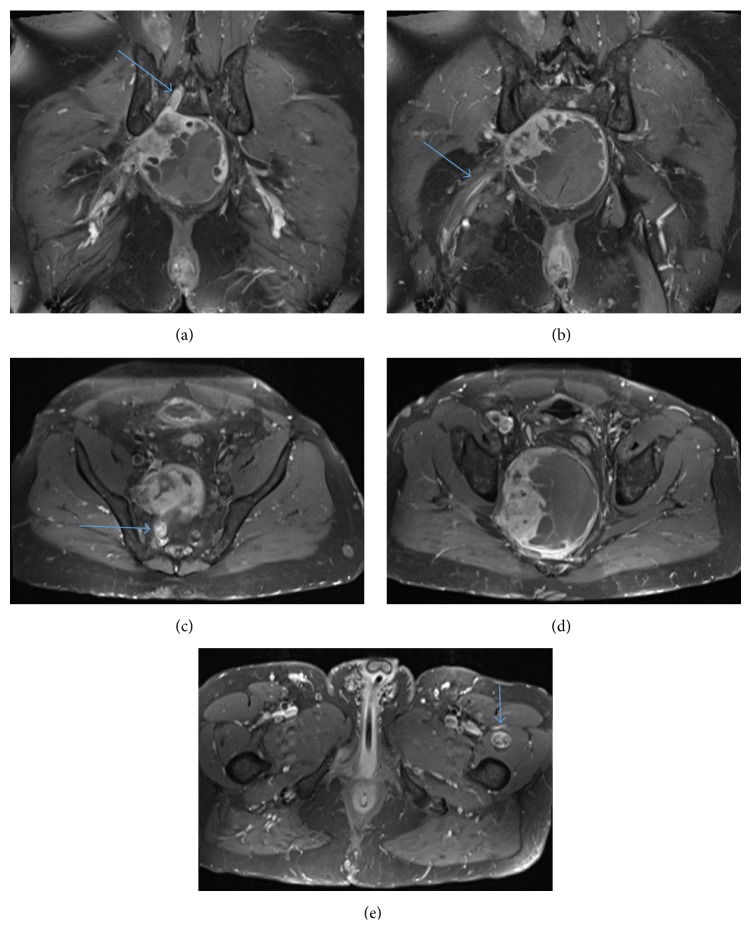
(a) Coronal T1 FS + contrast shows pelvic mass extending to the right S2 neuroforamen (arrow). (b) Coronal T1 FS + contrast shows pelvic mass (arrow points to right sciatic nerve). (c) Axial T1 FS + contrast shows pelvic mass extending to the right S2 neuroforamen (arrow). (d) Axial T1 FS + contrast shows pelvic mass. (e) Axial T1 FS + contrast shows left vastus medialis/intermedius muscle fibroma.

**Figure 6 fig6:**
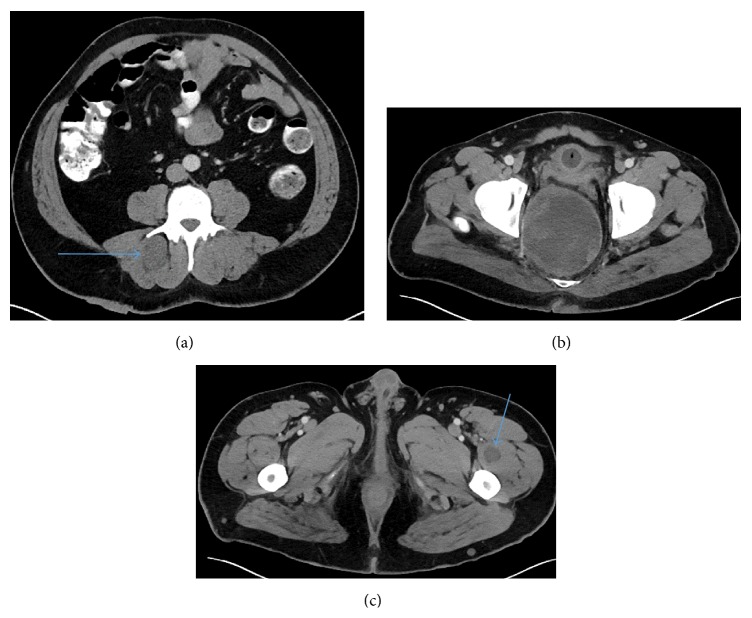
(a) Axial CT right shows right erector spinae/multifidus muscle neurofibroma (arrow); the attenuation of the mass is about the same to slightly less than that of muscle. (b) Axial CT shows heterogeneous pelvic mass. (c) Axial CT shows left vastus medialis/intermedius muscle fibroma (arrow); the attenuation of the mass is less than that of muscle.

**Figure 7:**
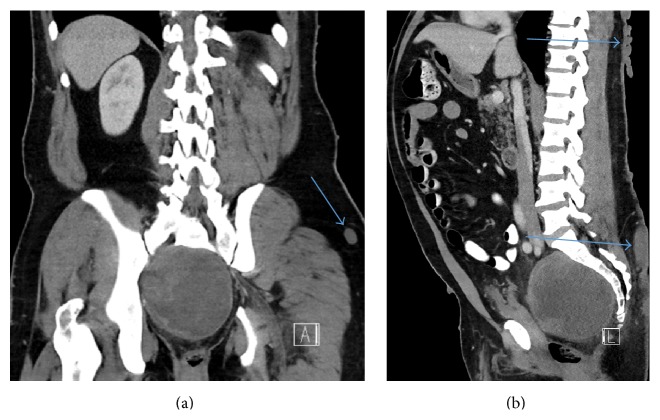
(a) Coronal CT shows heterogeneous pelvic mass and a left hip subcutaneous neurofibroma (arrow) which has the same attenuation as muscle. (b) Sagittal CT shows heterogeneous pelvic mass and numerous posterior subcutaneous neurofibromas (arrows) which have the same attenuation as muscle.

MRI of the lumbar spine showed a large mass extending anteriorly from the right S2 nerve root and in the posterior right lower back musculature at the level of L4-L5 and mildly enlarged left L2 ganglion and multiple skin neurofibromas. CT of the abdomen and pelvis showed a large complex pelvic mass measuring greater than 10 cm displaying mass effect on the posterior surface of the rectum arising from the right S2 neuroforamen. MRI of the abdomen and pelvis showed 9.6 × 9.8 cm complex cystic and solid enhancing mass in the right presacral space, extending from the right S2 neural foramen, displaces the rectum anteriorly and to the left (Figures 1 [Fig fig2] [Fig fig3] [Fig fig4] [Fig fig5] [Fig fig6]–7).

Pathology biopsy result showed malignant peripheral nerve sheath tumor (MPNST) with extensive necrosis.

## 3. Discussion

MPNST is a term that replaces malignant schwannoma, malignant neurofibroma, neurosarcoma, and neurofibrosarcoma. MPNSTs which account for 3–10% of all soft tissue sarcomas are rare and arise in proximity to large peripheral nerves. The most common location of these tumors is the trunk, extremities, or head and neck, being extremely rare in abdomen. The majority of MPNSTs are high grade sarcomas with high probability of recurrence and metastases. About half occur in patients with neurofibromatosis type 1 which have a poorer prognosis than MPNST in patients without neurofibromatosis type 1. Approximately two-thirds of MPNSTs result from malignant transformation of tumor associated with neurofibromatosis type 1. The overall lifetime risk of developing MPNST in neurofibromatosis type 1 patients is 8–13%. Neurofibromatosis type 1 is autosomal dominant disorder with highly variable phenotypic expression and natural history. The involvement of the abdomen and pelvis by neurofibromatosis type 1 is rare. Neurofibromatosis type 1 associated MPNST occurs in young adults (20–35 years of age) whereas sporadic MPNST occurs in middle age and older adults (peak: 5th and 6th decades). Pelvic peripheral nerve sheath tumors are very uncommon. Differential between benign peripheral nerve sheath tumor (BPNST) and MPNST is difficult because the former may be large and have marked atypical nuclei. The age range and size of BPNST and MPNST are 10–76 years (median, 54 years) and 4–20 cm (median size 9 cm) and 16–61 years (median, 34 years) and 9–17 cm (median, 12 cm), respectively. MPNSTs occurring in central locations such as the paraspinal region of the retroperitoneum have lower 5-year survival rates, higher recurrence rates, and higher frequency of metastasis compared to tumors in other parts of the body. MPNST may occur as solitary or multiple enlarged masses associated with major nerve trunks such as the brachial plexus, sacral plexus, and sciatic nerve and may be asymptomatic or present with various sensory and motor symptoms, including projected pain or compressing and infiltrating surrounding tissues and structures. About 10% of MPNSTs occur due to therapeutic or occupational radiation with a latent period of greater than 15 years [1–8].

BPNST is frequently asymptomatic whereas MPNST produces pain or neurological deficit. Pain at rest, tumor size, and duration of symptoms are of little value in differentiation between BPNST and MPNST. Faint motor weakness may be present in BPNST whereas severe motor weakness is seen exclusively in MPNST. MPNST may occur as solitary or multiple enlarged masses associated with major nerve trunks such as the brachial plexus, sacral plexus, and sciatic nerve and may be asymptomatic or present with various sensory and motor symptoms, including projected pain or compressing and infiltrating surrounding tissues and structures. The involvement of the abdomen and pelvis by neurofibromatosis type 1 is rare presenting with dysfunction and obstruction of genitourinary and gastrointestinal tracts. Patients with BPNST present with pain at rest (79%), sensory disturbance (53%), and faint-to-mild motor weakness (32%). The duration of the symptoms is 2–120 months (median, 12 months). All patients with MPNST have pain at rest (100%), some patients have sensory disturbance (73%), and other patients have faint-to-severe motor weakness (64%). Symptom duration was 3–96 months (median, 12 months) [4, 6].

The correct histological diagnosis of MPNST is made in about 17–41% of new patients, whereas about 52% were diagnosed as having another type of sarcoma, about 4% were classified as suspicious, and 4% represented false negatives due primarily to profound cytological overlap with other sarcomas. The diagnosis of MPNST is extremely difficult due to the lack of (1) conclusive immunohistochemistry or unique chromosomal anomaly, (2) universal distinctive histopathology, and (3) clinical criteria. The clinical, radiologic, and histologic presentation of MPNST is important in its diagnosis [7].

In the present case report, MPNST has the following: (1) MRI signal characteristics: high signal intensity: STIR, T2, and STIR > T2; intermediate signal intensity: PD FS, T1 FS, and PD FS > T1 FS; low signal intensity: T1, isointense to muscle; heterogeneous enhancement, whereas skin neurofibromas have high signal intensity: STIR, T2, and STIR > T2; intermediate signal intensity: T1; uniform enhancement; and (2) CT with contrast attenuation characteristics: heterogeneous attenuation; small lesions: attenuation lower than muscle; skin neurofibromas: attenuation same as muscle, with no significant enhancement. MRI is better than CT at detecting small lesions. CT did not show the smallest lesions but it is adequate for diagnosis if the patient cannot have MRI. The most sensitive MRI sequence for detection of MPNST is STIR. Heterogeneous hypointense signal on T1WI and heterogeneous hyperintense signal on T2WI are due to high water content of myxoid matrix or cystic or necrotic degeneration. Central areas of low signal intensity may be seen due to fibrosis. Post-IV contrast CT may show no contrast enhancement due to areas of necrosis, hemorrhage, or cystic degeneration. About 40–50% of BPNSTs may have central enhancement on CT corresponding to central zone of tightly packed cellular components (Antoni A) surrounded by hypocellular myxoid material (Antoni B) corresponding to target sign on MRI T-2 weighted images (central low signal intensity, peripheral high signal intensity). The central enhancement and target sign are rarely seen in MPNST. In large tumor, there is central necrosis or degeneration, with peripheral tumor enhancement. The borders in MPNST are frequently irregular and infiltrative borders may invade adjacent structures or destroy adjacent bones. CT and MRI cannot accurately differentiate between MPNST and BPNST because tumor heterogeneity, irregular or infiltrative borders, and bone erosion may be seen in both MPNST and BPNST [2, 4, 6].

MPNST is high grade tumor with high mitotic rate. However, histological diagnosis of MPNST is very difficult due to the lack of (1) histological and immunohistochemical markers specific for malignant peripheral nerve sheath tumor and (2) standardized diagnostic criteria. However, S-100 protein is seen in 50–90%, Leu-7 in 50%, and myelin basic protein in 40% and Ki-67 indices are seen in 5–65% of MPNSTs. Coinactivation of TP53 and Rb pathways is seen in 75% of MPNSTs [1, 3, 4].

The most common metastatic site of MPNST is the lung, followed by the bone, pleura, retroperitoneum, and subarachnoid space of the spine [1]. The patient had S1–S3 laminectomy and decompression of his spinal roots with a transsacral debulking of the presacral tumor. The patient had partial resection of the mass via anterior approach with about 80% of the tumor resected. The surgery was aborted due to significant bleeding requiring embolization for control. About 2 months later, the patient had a second surgery via posterior approach, but the whole tumor could not be completely resected. The patient suffered right ureteral cautery injury during the second surgery that required right ureteroureterostomy. Further surgery is planned after outside consultation. Nonsurgical treatment of MPNST is limited. The most effective treatment and important prognostic factor of MPNST are en bloc or wide surgical excision that includes surrounding nerves. However, complete resection of MPNST is rarely possible. Benign peripheral nerve sheath tumor can be resected without neurological deficit. Adjuvant radiotherapy may be used for local control. However, radiotherapy and chemotherapy appear to have little impact on the survival of patients with MPNST [1–3].

Survival of MPNST is associated with complete tumor resection. The overall 5-year survival rate is about 44–50% which is affected by the patient's age, size of tumor, location of tumor, and margins affecting survival. Tumor size is the most reliable independent prognostic factor, with larger tumors having worse prognosis. Tumors occurring in central locations such as the paraspinal region of the retroperitoneum have lower 5-year survival rates, higher recurrence rates, and higher frequency of metastasis compared to tumors in other parts of the body. Longer survival is associated with early diagnosis (due to improved imaging), aggressive treatment with complete surgical resection and neoadjuvant therapy, small tumor size (<5 cm), and presence of low grade components. Negative staining for S-100 is associated with better prognosis for completely resected tumors [1, 3].

## 4. Conclusion

A rare case of MPNST that produced urinary retention and bowel incontinence is presented that may aid clinicians in the diagnosis of this rare clinical entity. Motor weakness, central enhancement, and immunohistochemistry may assist in the diagnosis of MPNST and differentiation between BPNST and MPNST.
